# Structural Properties and Complexity of a New Network Class: Collatz Step Graphs

**DOI:** 10.1371/journal.pone.0056461

**Published:** 2013-02-19

**Authors:** Frank Emmert-Streib

**Affiliations:** Computational Biology and Machine Learning Laboratory, Center for Cancer Research and Cell Biology, School of Medicine, Dentistry and Biomedical Sciences, Faculty of Medicine, Health and Life Sciences, Queen’s University Belfast, Belfast, United Kingdom; Humboldt University, Germany

## Abstract

In this paper, we introduce a biologically inspired model to generate complex networks. In contrast to many other construction procedures for growing networks introduced so far, our method generates networks from one-dimensional symbol sequences that are related to the so called Collatz problem from number theory. The major purpose of the present paper is, first, to derive a symbol sequence from the Collatz problem, we call the *step sequence*, and investigate its structural properties. Second, we introduce a construction procedure for growing networks that is based on these *step sequences*. Third, we investigate the structural properties of this new network class including their finite scaling and asymptotic behavior of their complexity, average shortest path lengths and clustering coefficients. Interestingly, in contrast to many other network models including the *small-world* network from Watts & Strogatz, we find that CS graphs become ‘smaller’ with an increasing size.

## Introduction

The analysis of networks is a prospering field that spans many disciplines ranging from biology, mathematics and statistics to the social sciences [1]–[6]. Starting with the study of random networks [7], the interest of the community shifted in recent years to so called *complex networks* [8], [9]. In contrast to random networks, which show a Poisson distribution in the node degrees, many complex networks exhibit a power law distribution [8], [10]. The attention in complex networks can be at least partly attributed to the fact that they appear to be omnipresent in nature. This makes such networks not only interesting from a theoretical but also from a practical point of view [11]–[13].

The major purpose of the present paper is three-fold. First, we derive a symbol sequence from the Collatz problem, we call a *step sequence*. The Collatz problem [14], [15] is from number theory and it refers to the mathematical statement that starting from any natural number, the iterative application of a certain mathematical function leads always to the number 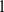, possibly, via intermediate natural numbers. Hence, the Collatz problem leads to the generation of one dimensional symbol sequences of natural numbers that all end at 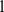. Second, we introduce a new construction procedure for growing networks that is based on the *step sequences* from the Collatz problem. For this reason we are calling the resulting networks CS (Collatz step) graphs. This will simulteneously lead to the definition of a new class of complex networks. Third, we investigate the structural complexity and the scaling behavior of *step sequences* and CS graphs, including an estimate for the asymptotic complexity of CS graphs.

In contrast to many other generation procedures for growing networks introduced so far [2], [16]–[18], our method constructs networks from one-dimensional symbol sequences that are related to the Collatz problem [14], [15]. We would like to emphasize that we are not the first to map one-dimensional objects to networks. For instance, for time series data this has been done in [19]–[24] and in [25], [26] prime number related networks have been constructed. An even older example for such a construction principle can be found in [15] constructing a so called *Collatz graph*. In this paper, we tie on the work in [15], however, constructing networks from a different type of sequences that can be derived from the Collatz problem. Interestingly, we will show that *Collatz graphs* and CS graphs are entirely different and we will argue that this is related to the differences in the underlying sequences, respectively the difference in their complexity.

In this context it is interesting to note that the conjecture made by the Collatz problem is to date mathematically unproven. However, it has been numerically verified up to natural numbers as high as 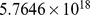 [27]. For this reason it can be assumed that this intricate problem is capable of generating symbol sequences which are truly complex.

The motivation for our network model is based on the working mechanism of a biological cell. In a cell, a linear symbol sequence, the DNA, is transcribed into mRNAs and then translated into proteins which form protein interaction, signaling or other types of gene networks [28]–[30]. That means our construction procedure, which may appear unconventional at first if compared to well known mathematical mechanisms to generate growing networks [17], [18], is in fact employed by nature. In addition to this, we want to note that protein interaction networks and signaling networks can be considered as complex, not only because they exhibit a power law distribution in their degrees, but because these networks form an integral part of the functioning of living cells. Further, studies have shown that also the DNA sequence itself can be considered as a complex symbol sequence [31], [32]. This suggests that the complexity of the DNA sequence carries over to the complexity of gene networks. In this context it appear plausible to assume that not any arbitraray DNA sequence leads to complex (gene) networks but the DNA sequence needs to be complex itself. From an abstract point of view, we base our network model on these observations by exploiting the mechanism of the biological counterpart. Specifically, we use symbol sequences that are related to the Collatz problem [14], [15] as starting point for our model.

This paper is organized as follows. In the next section, we introduce all mathematical definitions and preliminaries we need for our analysis. Further, we define *step sequences*, CS graphs and our procedure to generate growing networks. In the results section, we present our analysis of *step sequences* and CS graphs, studying their complexity and scaling behavior. Further, we provide estimates for the asymptotic complexity, average shortest path lengths and clustering coefficients of CS graphs. This paper finish with a discussion and conclusions.

## Methods

In this section we introduce the basic definitions and notations we need to introduce our network model. This includes a brief description of the Collatz problem as far as it is necessary for our analysis.

### Basic Definitions: Collatz Problem, Sequence and Graph

A Collatz sequence is defined for every natural number 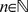 according to the iterative application of the following mapping.
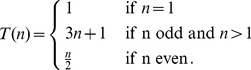
(1)


**Figure 1:**
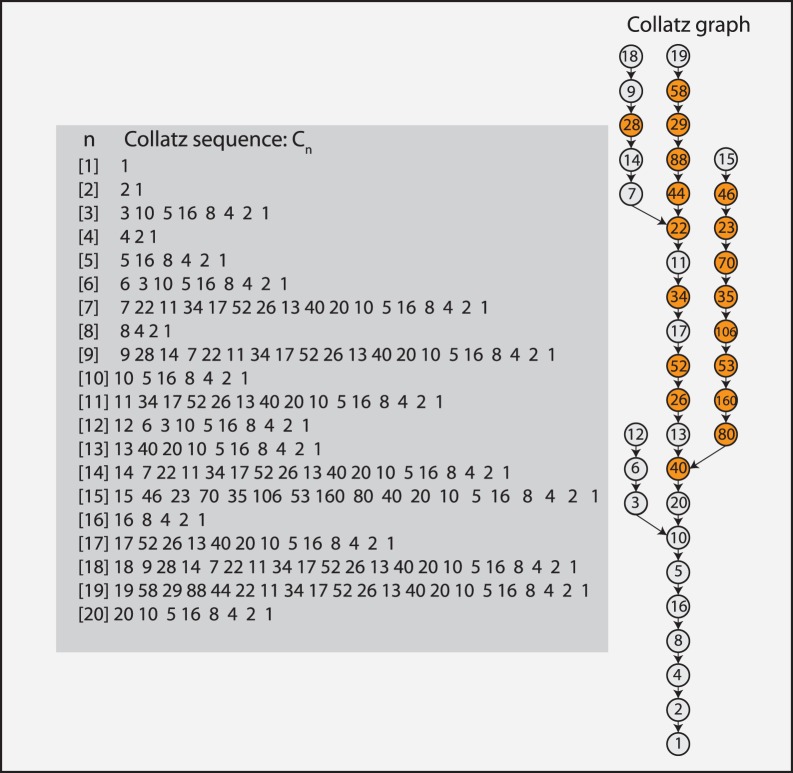
Collatz sequences and Collatz graph. Left: Examples of Collatz sequences, *C_n_*, for the first 20 natural numbers. Right: A network representaion of these sequences is called the Collatz graph.

For example, for 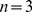 we obtain the sequence 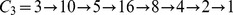. This sequence is called the *Collatz sequence* for 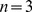. Further examples for the first 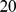 natural numbers can be found in Fig. 1. That mean the iterative application of Eqn. 1 maps the natural number 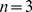 after 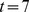 steps to 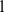, i.e., 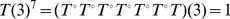. Further application of Eqn. 1 cannot lead to other results because ‘
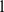
’ is a fixed point of the above mapping. If one considers the natural numbers as states of the transformation, the above sequence can be visualized in the state space by consecutive mappings between adjacent states. A visualization of the state space for the first 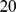 Collatz sequences is shown in Fig. 1. Due to the fact that the state space is discrete, its representation corresponds to a network. This network has been termed the *Collatz graph* [15].

We would like to note that the number of elements in the state space corresponds to the number of natural numbers that are *traversed* from the initial natural number 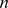 to 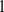. However, we would like to emphasize that these elements do not necessarily correspond to the consecutive natural numbers 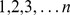. If a state space of a set of sequences, e.g., 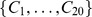, is considered than the number of elements in this state space is the union of the elements of the individual sequences, i.e., 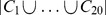. It is interesting to note that there are two types of states, which can be naturally distinguished from each other. The first type of states consists of the natural numbers, 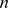, that were used as initial value to generate a Collatz sequence, 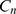, whereas the second type of states are states with values 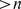. In order to visualize this, in Fig. 1, we show states of the first type in gray color and states of the second type in orange.

Based on the above mapping in Eqn. 1, Lothar Collatz conjectured in 1937 that every natural number, 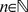, will be mapped to 1 by its iterative application. This is also know as the 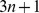 conjecture or the Syracuse problem [33], [34]. To date, this conjecture remains mathematically unproven, however, numerically it has been verified up to 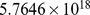 [27]. In this paper we will not be concerned with the proof of this conjecture, but with the the disposition of the *step sequence*, that can be derived from the Collatz problem, as discussed in the next section.

### Step Sequence and CS Graph

In addition to the state space generated by the application of, 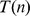, there are other, different symbol sequences one can obtain that are also based on 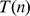. In the following, we will derive such a symbol sequence.

In order to define our new symbol sequence properly, we need to introduce two functions, 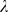 and 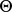. The first function, 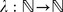,
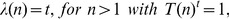
(2)defines a mapping from a natural number 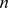 to the number of iteration steps 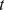 it takes 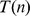 to map to 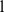. For this reason, we call 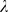 the *step function*.

Based on 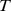 and 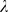, we define a second mapping 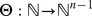 by
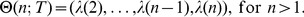
(3)


**Figure 2:**
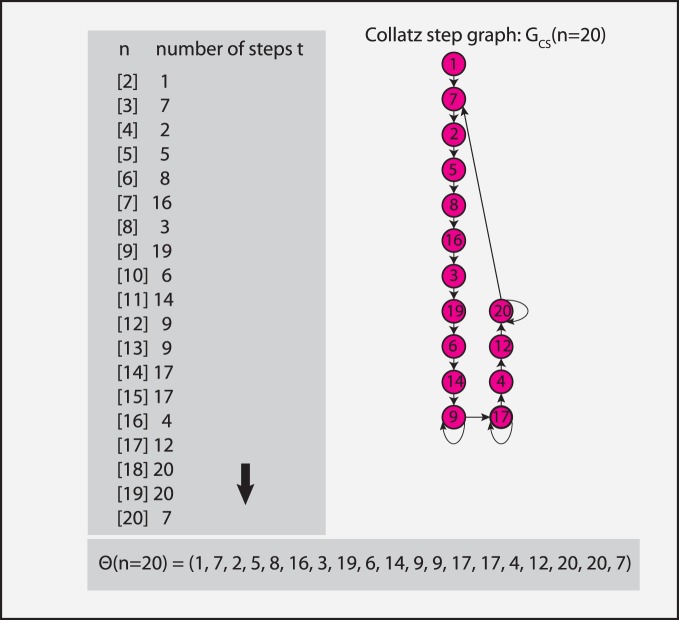
Collatz step graph. Left: The results of the step function as a function of *n*. Bottom: The *step sequence* 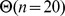 that is based on the results of the step function. Right: From 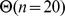 a CS graph can be constructed.

That means 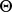 is a vector valued function whose components are indexed by 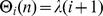 for 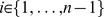. The function 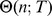 allows to generate symbol sequences of length 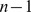 whose elements assume values in 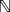. For example, 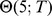 generates the sequence 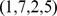. Further examples can be found in Fig. 2. We call a symbol sequence generated by 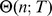 a *step sequence* because the value of each component 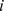 of this sequence corresponds to the number of iteration steps the mapping 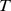 needs to be applied to map 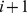 to 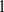. In the following, we write 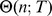 briefly as 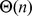.

For a *step sequence* 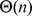 there is a natural representation in form of a network. More precisely, let the different elements of a *step sequence* correspond to the vertices 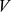 of a network and the edges 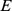 are defined by consecutive subsequences of length 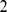 of the *step sequence*.

### Definition: CS Graph

We define a *Collatz step graph*, briefly called CS graph and denoted by 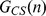, for a *step sequence* 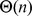 in the following way.

Vertex set:
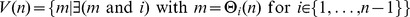
(4)


Edge set:
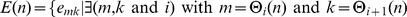


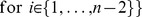
(5)


Weighted edge set:
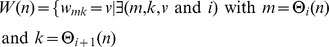


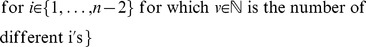
(6)


The networks defined in this way are directed and weighted, and the edge weights assume values in 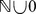, whereas 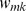 corresponds to the number of times the state 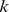 follows state 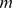 on the *step sequence* 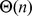. On a mathematical note, we want to remark that due to the fact that a *Collatz step graph* is constructed based on a *step sequence* 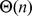, the resulting network is indexed by 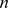. That means for each 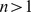 one obtains a CS graph 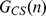.

**Figure 9:**
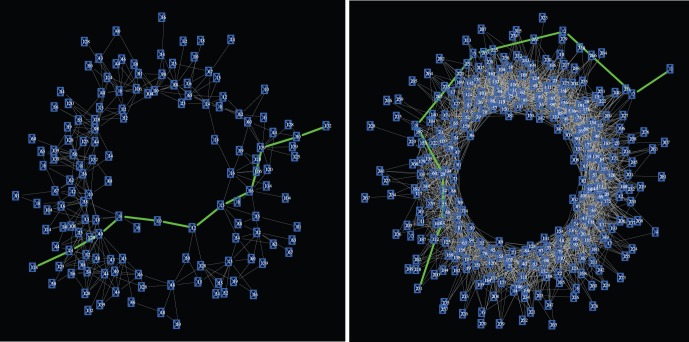
CS graphs. Left: CS graph, 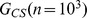, with 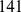 nodes, 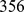 edges and a diameter of 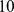. Right: CS graph, 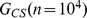, with 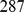 nodes, 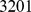 edges and a diameter of 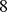.

In Fig. 2 we visualize 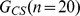 for the *step sequence*,
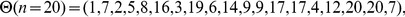
(7)shown on the bottom in the Fig. 2. Due to the fact that the vertices in this network correspond to the number of steps to map a certain natural number to 1, rather than to the natural numbers 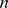 themselves nor to the intermediate numbers as for the Collatz sequence, we emphasize this distinction in the meaning of these elements in a CS graph by a different node color, compared the Collatz graph in Fig. 1. In Fig. 9 we show more complex CS graphs for 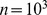 (left) and 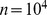 (right). In this case, the networks consist of 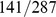 nodes and 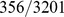 edges.

Descriptively, the definition of a CS graph can be visualized by traversing a *step sequence* from the first element to the last element, corresponding to the vertices in the network, and by connecting consecutive elements with an edge. If an element of the *step sequence* appeared already at an earlier step, no new vertex is included, but only an edge to this vertex.

### Growing Network Model

Using the definitions from the previous section one can alternatively formulate a **g**rowing network **m**odel for **CS** graphs, called GMCS. That means this model grows a CS graph, 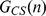, as a function of natural numbers. Algorithm 1 describes this model formally. We formulate the GMCS in terms of the *step sequence* 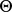, however, we would like to note that an equivalent formulation can be achieved by using the *step function* 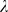 instead, because according to Eqn. 3 one can write,
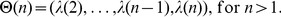
(8)


Interestingly, in contrast to many other models for growing networks, e.g., random networks or scale-free networks [2], [16]–[18], the construction principle of CS graphs is different. The difference to these models is that an one-dimensional symbol sequence, given by 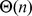, is used to determine the growth of the network. In the results section, we investigate different structural aspects of the *step sequence* to the resulting CS graphs. Another difference between our model and, e.g., [2], [16]–[18] is that for a fixed 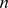 one obtains always the exact same graph 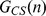. This is due to the fact that the underlying *step function* is deterministically formed. However, we will demonstrate that the generated networks exhibit nevertheless an astonishing complexity.

Availability: An R implementation of GMCS is available from *The Comprehensive R Archive Network* (CRAN; http://cran.r-project.org/).

## Results

In the following, we study, first, characteristics of the *step sequence* and its scaling behavior. Then we investigate structural features of CS graphs, their scaling behavior and the complexity of these networks.

**Algorithm 1** GMCS: Growing network model for CS graphs.

1: Given: 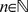 with 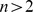.

2: Initialize: 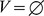, 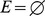, 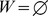.

3: Calculate: 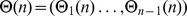.

4: 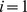.

5: 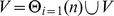.

6: while 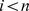 **do.**

7: **if** 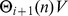 **then.**

8: 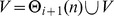.

9: **end if.**

10: **if** 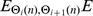 **then.**

11: 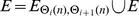.

12: **end if.**

13: **if** 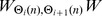 **then.**

14: 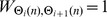.

15: 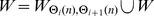.

16: **else.**

17: 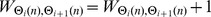.

18: **end if.**

19: 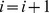.

20: **end while.**

21: Return: CS graph with 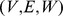.

### Properties and Scaling of the Step Sequence

**Figure 3:**
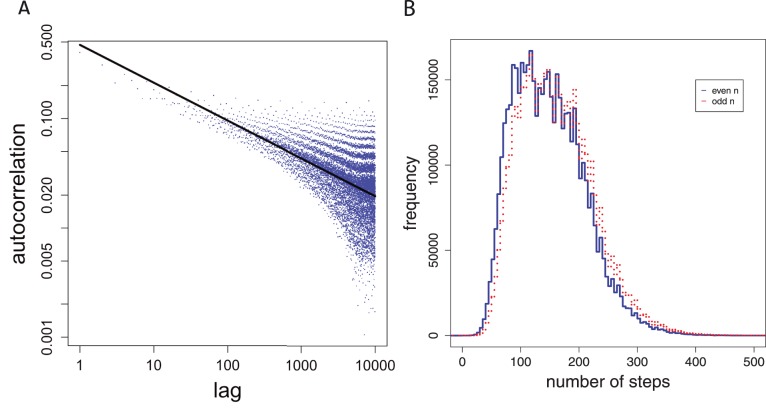
Properties of step sequences. A: Autocorrelation function, 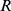, of the Collatz step sequence. B: Histogram for even and odd sequence elements of 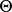.

In order to quantify the behavior of the *step sequence* 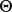 we, first, calculate the autocorrelation function, 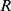, for a *step sequence* of length 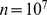. Fig. 3 A shows a visualization of 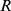 in a double logarithmic plot. There, one can see that up to 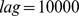 there is still a relatively high correlation (higher than expected for a random sequence) between the shifted sequence indicating the presence of a long term memory. Usually, the presence of a long term memory in a symbol sequence is assumed as an indicator for the complexity of the sequence [35], [36]. We quantify this observation by performing a linear regression of the logarithm of the autocorrelation function and the *lag*,
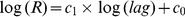
(9)

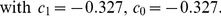
(10)


The scale-free nature of the autocorrelation function, 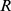, provides quantitative evidence for the complex nature of the *step sequence* 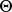.

Another indicator for the complexity of 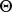 can be revealed with the help of two histograms obtained for even and odd elements of the *step sequence*. More precisely, we define for an even natural number 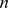 the following two sequences,
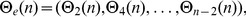
(11)

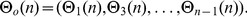
(12)


The histograms for 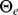 and 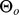 are shown in Fig. 3 B. One can see that both histograms can be clearly distinguished from each other indicating subtle differences between the odd and even elements of 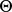. A possible explanation for this effect can be found in the asymmetric construction of the Collatz sequence that is based on 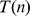, given in Eqn. 1, because this mapping treats even and odd numbers of 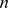 differently. However, this behavior is not trivial, because the asymmetry in even and odd sequence elements is not present in the autocorrelation function, 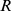, if the sequences 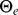 and 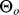 are used for its calculation (results not shown).

The last property of 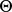 we study is the scaling behavior of the mean number of steps, 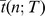, it takes the mapping 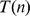 to converge, which corresponds to the time to reach its fixed-point. More precisely, we define,
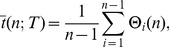
(13)and perform a linear regression for 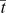 on the logarithm of the length 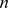 of the *step sequence* (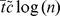). This gives a scaling factor of 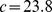 with a p-value of 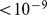. From our result follows that the scaling of 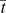 is well approximated by the logarithmic growth in 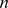, which means that even for natural numbers as large as 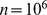, the mapping 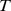 converges in average in only about 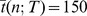 iteration steps.

### Complexity and Scaling of CS Graphs

**Figure 4:**
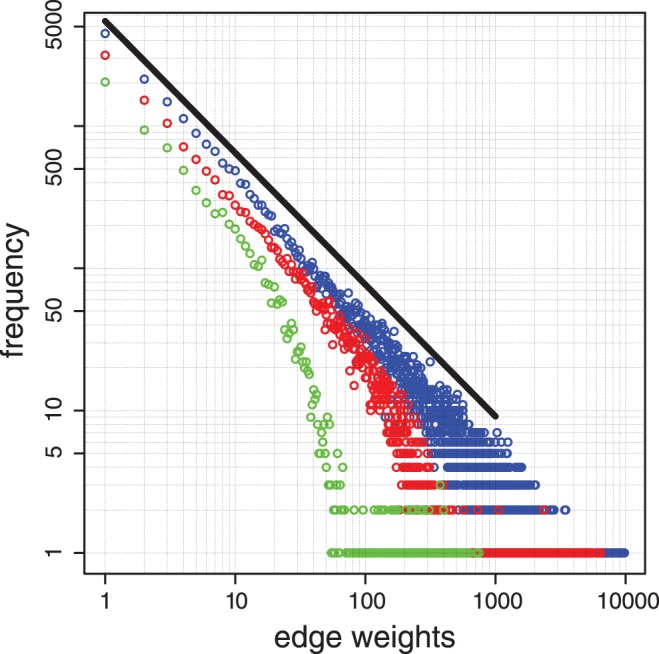
Power law scaling of the edge weights in CS graphs. The color corresponds to different sizes of 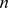 with 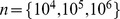 (green, red, blue).

Now we turn to the investigation of structural properties of CS graphs. We start by studying the scaling of the edge weights. The results of this scaling for three different CS graphs obtained for 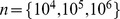 (green, red, blue) are shown in the double logarithmic plot in Fig. 4. More precisely, this figure shows the frequency distribution of the edge weights of the CS graphs. Here, the edge weights correspond to the number of times these edge have been visited when traversing the *step sequence* from its first to its last element, as defined in Eqn. 6. One can see that all three networks follow asymptotically a power law with nearly the same exponent of 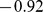. Further, the change toward larger values of 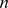 effects only the range of the power law behavior, but not its exponent.

**Figure 5:**
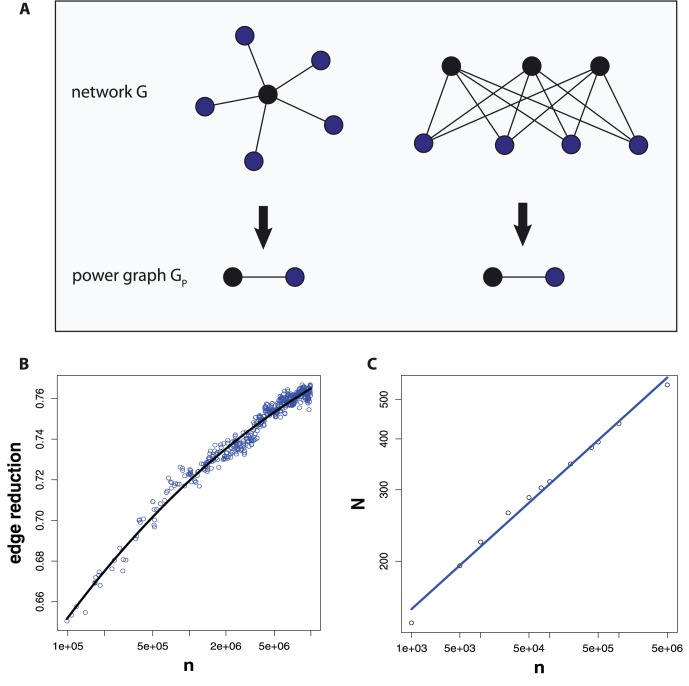
A: Examples of lossless transformations underlying the construction of power graphs and the *edge reduction* measure. B: The *edge reduction* of CS graphs as a function of the length of the *step sequence*. C: Scaling of the size, 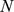, of CS graphs as a function of the length, 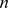, of the *step sequence*.

Next, we study the finite and asymptotic structural complexity of the CS graphs. There are many measures that have been suggested over the past decades to quantify the complexity of networks [37]–[43]. However, currently, there is no generally accepted gold standard available that is comparable to the Kolmogorov complexity for symbol sequences [44]–[46]. For our specific context and the construction of the CS graphs based on the symbol sequence 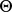, a recently introduced measure termed *edge reduction* [47] seems to be most appropriate. The *edge reduction* is based on so called power graphs 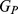 which are obtained from an underlying network 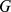 by grouping nodes and edges that are similar to each other. For example, if a vertex is star-like connected to a group of other vertices, then the group of nodes appears as one node in a power graph, see Fig. 5 A. Another example is a bipartite connection of two groups of nodes, as shown in the right figure in Fig. 5 A.

The measure itself is defined by.
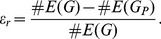
(14)


Here 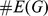 corresponds to the number of edges in network 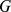 and 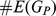 is the numbers of edges in the power graph 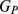. Hence, 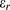 measures the reduction in the number of edges in the power graph compared to 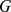. It is important to note that power graphs form a lossless representation of the original network 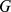, which reduces the network complexity by explicitly representing reoccurring network substructures. The underlying construction principle of *edge reduction* reminds of self-similarity, observed, e.g., in fractal structures [48], [49]. For this reason we consider *edge reduction* as an intuitively plausible quantification of the structural complexity of networks.

In order to study the *edge reduction* of CS graphs numerically, we generate a random sample of 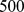 natural numbers 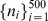 from the interval 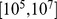 and construct for these numbers their corresponding CS graphs 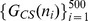. Then we determine for each CS graph its *edge reduction*, 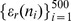. Because the values of 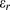 are restricted to the closed interval between zero and one, the *edge reduction* cannot grow infinitely, but needs to converge asymptotically for large values of 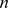. For this reason we fit a logistic function [50], [51],
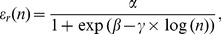
(15)to these values, modeling the growth of 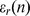 with respect to 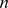. The results are shown in Fig. 5 B. The parameters we obtain from a nonlinear least square fit are shown in table 0. All parameters are highly significant as indicated by very low p-values. We use this result to predict the asymptotic *edge reduction* for 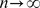. From Eqn. 15 we obtain



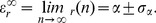
(16)That means the limiting *edge reduction* of a CS graph is 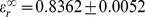.

**Figure 6:**
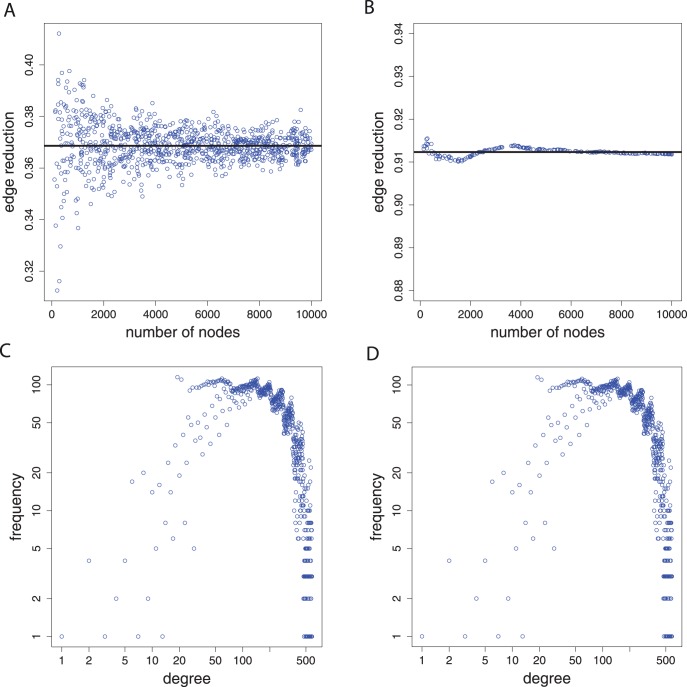
The *edge reduction* for random networks (A) and regular trees (B). The x-axis gives the number of nodes in these networks. Figure C and D show the in- and out-degree distribution of a CS graph for 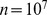.

To contrast this result with random and highly structured (simple) networks we calculate the *edge reduction* measure also for 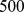 random networks and 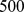 trees. The results from this are shown in Fig. 6 A and B. We would like to note that the obtained *edge reduction* values for random networks and trees are much smaller respectively larger than for CS graphs. These results are intuitively plausible because random objects are difficult to compress, whereas simple object compress easily. Also, it is generally observed that complex objects fall between random and simple objects [52], as is the case for CS graphs.

The second note we would like to make relates to the time scale it take the random networks and the trees to reach a steady-state value. As one can see from Fig. 6 A and B, the random networks and the trees reach for about 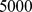 nodes in a network values that fluctuate around their corresponding mean values, indicated as black lines. The CS graphs show for *step sequences* of length up to 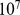 still a tendency to increase their 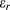 values. However, we would like to point out that the shown x-axes relate to different variables. Whereas in Fig. 6 A and B the x-axes correspond to the number of nodes in a network, in Fig. 5 B it indexes the length of the *step sequence*. To relate both scales with each other to obtain a fair comparison we, first, estimate the length of the *step sequence* for which the *edge reduction* of CS graphs is close to its limiting value 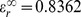. Inverting Eqn. 15 and using 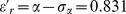 as such an approximation, because this value is within one standard deviation of the estimated limiting value, we obtain.
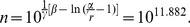
(17)


Then, we perform a linear regression for the number of nodes 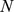 in CS graphs and the length of the *step sequence* 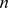, in a double logarithmic scale (see Fig. 5 C). The result of this is given by
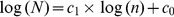
(18)

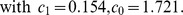



From this, we estimate the number of nodes in a CS graph that results from a *step sequence* of length 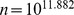, as predicted above. Using Eqn. 18 this gives 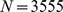 nodes. Interestingly, this number is slightly smaller than for the random networks and trees. However, the order of magnitude of the node sizes of all three network types, random networks, trees and CS graphs, is of comparable order.

The next question we address relates to the connectivity structure of CS graphs. There is the general *misbelief* that complex networks should show a power law behavior in their degree distributions [8], [10]. In Fig. 6 C and D we show the in- and out-degree distribution of a CS graph obtained for 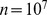. As one can see, the shown distributions are not scale-free, although, there is a very narrow region toward high degrees which might develop into a power law for an increasing size 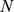 of the network. Hence, despite the fact that many types of complex networks exhibit a scale-free distribution in their degrees this is no necessary condition to constitute a complex network. Another example for this are the well-known *small-world* networks [5], [53] that do also not have a power law degree distribution.

**Figure 7:**
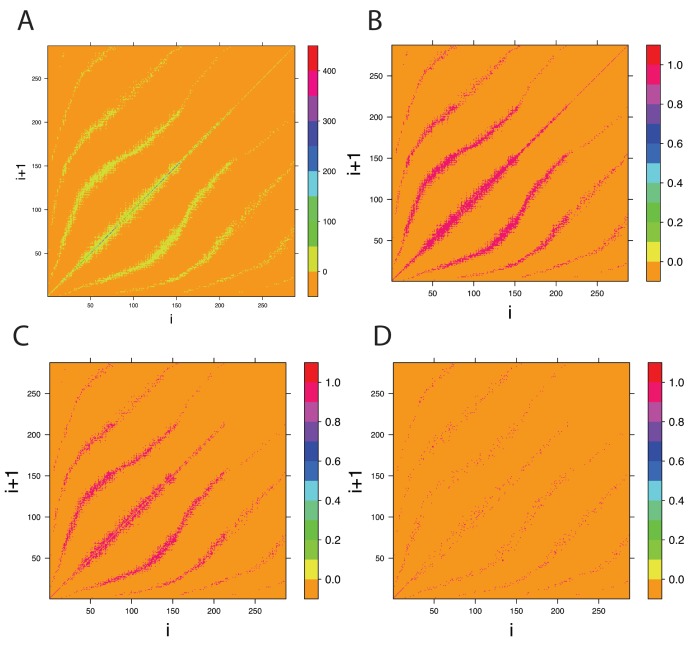
CS graph with 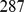 nodes obtained for 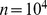. A: Adjacency matrix 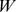. B: Binary adjacency matrix 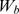. C: Non-equal elements 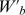. D: Non-symmetric elements 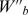.

Another interesting property of CS graphs is their structural connectivity pattern. In Fig. 7 we show different transformations of the adjacency matrix 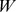 of the CS graph, 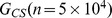. Figure A shows the adjacency matrix, 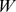, itself. Due to the fact that a CS graph is a weighted network we use different colors to emphasize different weights. Figure B shows a binary transformation (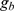) of 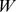, which maps non-zero elements to one and leaves zero elements unchanged, i.e.,
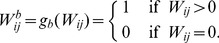
(19)


The resulting network, 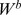, looks similar to, 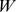, which can be attributed to the fact that most edge weights of 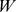 are of a comparable size. This is also supported by the exponential distribution of the in- and out-degrees, shown in Fig. 6.

In Figure 7 C we emphasize non-symmetric elements in 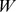. That means, we apply the following transformation:
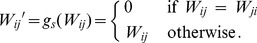
(20)


Then, we transform 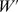 to a binary matrix 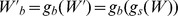, as described above. Figure 7 C shows 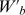. We are applying this transformation in order to demonstrate that the visual impression from Fig. A that 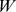 is symmetric, is not true. If 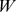 would be a symmetric matrix Fig. C would look quite different to Fig. A, because 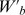 would correspond to the zero matrix.

If we consider a more general transformation than in Eqn. 20 considering the components of 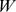 as similar when they are both larger than zero, but not necessarily equal,
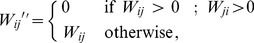
(21)we obtain a matrix 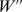. Interestingly, also the binary matrix 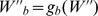 is not a zero matrix, as can be seen in Fig. D. This establishes that CS graphs are pseudo-symmetric in the sense that they are strictly considered not symmetric but, nevertheless, appear to be symmetric, as can be seen in Fig. 7. Overall, our results suggest that the general connectivity structure of the CS graphs is intricate and robust against different transformations.

### Average Shortest Path Lengths and Clustering Coefficients

Next, we investigate the scaling of the average shortest path lengths, 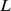, in CS graphs. Here, the average shortest path length is defined as the average over all shortest paths of all pairs of nodes in a network. In the following, we perform such an analysis for undirected and directed CS graphs, whereas the undirected CS graphs are obtained from directed CS graphs by neglecting the directionality of edges.

**Figure 8:**
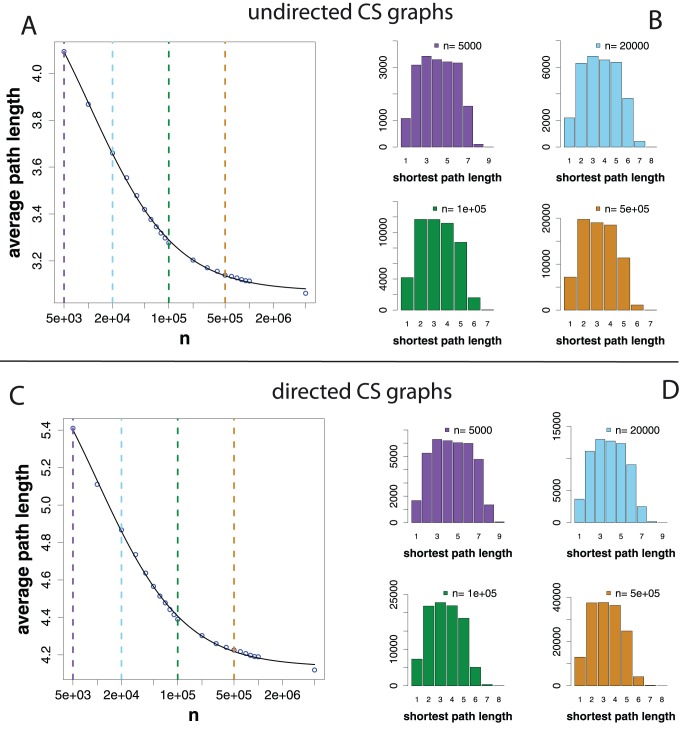
Average shortest path lengths. A: Scaling of the average path length in dependence on the size of the *step sequence* 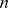. B: Histograms of the shortest path lengths for four different values of 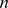. The colors correspond to the vertical lines in Fig. A.

Our results are shown in Fig. 8 A and C. Due to the fact that the average shortest path lengths decay for the undirected and directed CS graphs, we fit a nonlinear decay function,
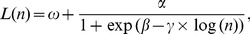
(22)in order to determine their finite scaling and asymptotic behavior. In Eqn. 22, 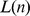, corresponds to the average shortest path length in dependence on the size of the *step sequence*, 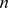. Table 2 gives the fitted parameter values for Eqn. 22. We would like to emphasize that due to the decay of the average shortest path lengths, the sign of 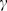 is now negative, in contrast to the results for the growth model, given in table 1.

**Table 1:** The parameters of a logistic function obtained from a nonlinear regression.

parameter name	value	SD	p-value
*α*	0.8362	0.0052	<10^–16^
*β*	1.5029	0.1012	<10^–16^
*γ*	0.5535	0.0245	<10^–16^

**Table 2:** The parameters of the logistic decay function obtained from a nonlinear regression.

	undirected			directed		
parameter name	value	SD	p-value	value	SD	p-value
*α*	1.590	0.089	1.94⋅10^–12^	2.000	0.143	9.33⋅10^–11^
*β*	–7.451	0.424	2.47⋅10^–12^	–7.355	0.526	9.42⋅10^–11^
*γ*	–1.856	0.079	2.58⋅10^–14^	1.841	0.099	9.42⋅10^–13^
*ω*	3.069	0.006	<2⋅10^–16^	4.134	0.009	<2⋅10^–16^

From Eqn. 22, we predict the asymptotic average shortest path lengths for 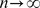. From Eqn. 22 we obtain,
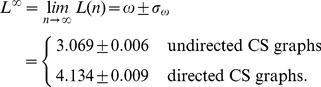
(23)


It is very interesting to see that 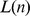 decays for larger values of 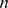 and, hence, the size of the CS graphs. This is quite different to the behavior of random, scale-free and *small-world* networks, because all these models *increase* their path length with increasing size of the network. More precisely, the scaling for the average shortest path length for these three network models is [8], [18]:
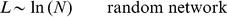
(24)

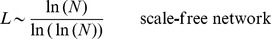
(25)

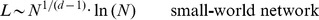
(26)


An explanation for this behavior of CS graphs can be given with the help of the distribution of shortest path lengths, as shown in Fig. 8 B and D. In these figures, we show four different histograms for undirected and directed network, each for a different size 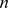 of the *step sequence*, as indicated by the color of these histograms, which correspond to the vertical lines in the Fig. 8 A and C. From the histograms one can see that the diameter of the CS graphs, which is the maximal length of all shortest paths, decreases slightly from 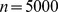 to 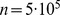. Further, the absolute number of shortest paths increases strongly, as can be seen from the increasing values of the y-axes. Taken together, this results in an overall decrease in the average length of the shortest paths.

In Fig. 9 we show a visualization of two undirected CS graphs, which makes this behavior even more clear. For reasons of clarity of the presentation, we removed loop connections. The left figure shows the CS graph, 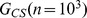, with 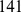 nodes and 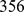 edges that has been generated from a *step sequence* of length 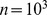, whereas the right figure shows the CS graph, 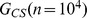, with 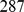 nodes and 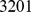 edges generated with 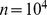. It is interesting to observe that both network structures are similar to a torus, forming a kind of ring. For increasing 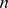 the torus gets more dense in the sense that there are more nodes and edges around the ring, however, the overall structure is conserved. The two figures in 9 include also two shortest paths of maximal length, corresponding to the diameter of the networks, shown in green. The diameter of 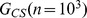 is 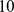 and the diameter of 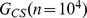 is 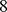. Considering Fig. 9 together with the Figs. 8 B and D, the shown histograms can be interpreted easily. First, the maximal shortest path length in the Figs. 8 B and D corresponds to the diameter of the corresponding networks, which decreases slightly. Second, the increasing number of shortest path lengths from 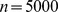 to 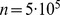 (see scale of the y-axes) is caused by the increasing density of the nodes on the tori. Third, averaging over all shortest path lengths leads to decreasing path lengths from small to high values of 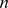. This is not only because of a decreasing diameter of the CS graphs but also due to the largely increasing number of short path lengths (for instance shortest paths of length 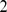).

The shown CS graphs in Fig. 9 are undirected. However, for directed CS graphs we obtain qualitatively similar results. This can also be seen from the Figs. 8 C and D. The only quantitative difference between undirected and directed CS graphs is that the observed (average) path lengths are larger for directed CS graphs.

Finally, we study the clustering coefficients of CS graphs and investigate if these networks exhibit a ‘small-world’ network characteristics. The (global) clustering coefficient 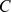, also called transitivity [4], is defined as
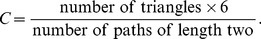
(27)


**Figure 10:**
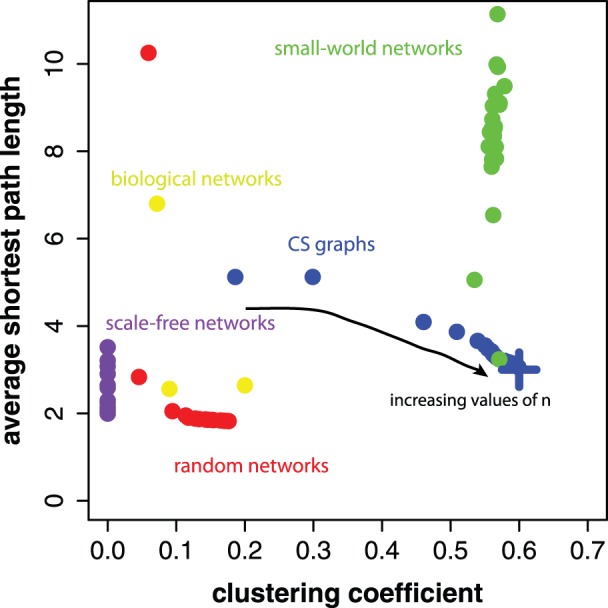
Average shortest path lengths and clustering coefficients. CS graphs (blue), random (red), small-world (green), scale-free (purple) and biological (yellow) networks. The blue cross indicates the limiting value for CS graphs.

In Fig. 10, we show for 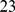 different CS graphs, generated from 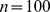 to 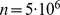, their average shortest path lengths in dependence on the clustering coefficients (in blue). For reasons of reference, we include in this figure also random (red), small-world (green), scale-free (purple) and biological (green) networks. The random networks have been generated with an Erdös-Rény model [2]. More precisely, for each CS graph, we generate one Erdös-Rény random network with the same size (number of nodes) and the same number of edges. The small-world networks have been generated with the algorithm of Watts and Strogatz with a rewiring probability of 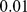 and three neighbors in an one-dimensional model [5]. The scale-free networks have been generated with the preferential attachment algorithm of [16] and randomly selected exponents between 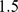 and 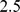. Also the size of small-world and scale-free networks corresponds to the size of the CS graphs. The three biological networks correspond to a metabolic network, a PPI network from yeast and a neural network from *C. Elegans* [9], [54]–[56]. One can see that with an increasing length of the *step sequence*, the clustering coefficients of the CS graphs increases whereas the average shortest path lengths decrease simultaneously slightly. Overall, we find that the clustering coefficients are generally larger than of random and biological networks, and comparable with that of small-world networks. This suggest, that CS graphs could be considered as *small-world* networks, because they have a high clustering coefficient and a small average path length. We included the biological networks in this figure to show that CS graphs are also remarkably different to natural networks that represent complex mixtures of scale-free, random and small-world networks, as biological networks. Further, despite the fact that the three biological networks consist up to thousands of nodes, and not hundreds as all other networks, this imbalance does not lead to an increasing similarity to CS graphs. In contrast, the larger the CS graphs, the more different they become.

As the limiting value of the clustering coefficient, fitting a logistic growth function as above (see Eqn. 15), we estimate.
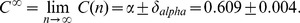
(28)


The blue cross in Fig. 10 indicates the limiting clustering coefficient and average shortest path length (see Eqn. 23) for CS graphs.

## Discussion

The present paper introduced and studied a novel network class. More precisely, we, first, defined and derived a one-dimensional symbol sequence from the Collatz problem [14], [15], we called the *step sequence*. From investigating its structural properties, we found that a *step sequence* exhibits a complex behavior due to the presence of a long term memory. Second, based on *step sequences*, we introduced a construction procedure for growing networks and called the resulting networks CS graphs. Due to the explicit connection to one-dimensional symbol sequence our construction procedure is distinct to many other well-known growing network models [2], [16]–[18]. Third, we investigated the finite scaling and the asymptotic behavior of structural properties of CS graphs. More specifically, we found that despite the fact that CS graphs do not exhibit a scale-free distribution in their degrees, their edge weights follow a power law. Moreover, we demonstrated that the values of the *edge reduction* of CS graphs, which provides a measure for the structural complexity of networks, are situated between the values observed for random (random networks) and simple (trees) structures. This holds for finite values of 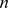 as well as asymptotically and, hence, provides evidence that CS graphs possess a complex network structure. This is also supported by our investigation revealing the pseudo-symmetric appearance of CS graphs. It is a well-know behavior that complex objects fall between random (chaotic) and simple (ordered) structures and it has been observed for a multitude of different systems, e.g., for cellular automata or Boolean networks [57]–[59].

In addition, we found that the finite scaling of the average shortest path lengths of CS graphs can be approximated by a logistic decay function. This results in the curious behavior that growing CS graphs become ‘smaller’ with respect to their average shortest path length. Interestingly, despite the seeming similarity of CS graphs and *small-world* networks generated with the Watts & Strogatz model [5] (see Fig. 9), this characteristics makes them distinct from each other.

We would like to re-emphasize that the CS graphs investigated in this paper, are constructed from one-dimensional symbol sequences (Collatz step sequences) generated by the iterative mapping of natural numbers, governed by Eqn. 1, starting from a natural number 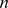. For this reason, we consider it natural to present our result about the structural properties of CS graphs with respect to 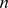. Instead, usually, properties of networks are studied in dependence on the size of the networks (number of nodes) 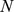. First, we would like to note that there is a simple scaling between 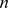 and 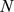, shown in Fig. 5, which allows to convert the results. Second, all statements in this paper are independent of particular values of 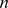 and, hence, of 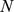 (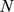 follows from 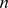, not vice verse). This includes also the asymptotic results. Third, other network models are *not* constructed from one-dimensional symbol sequences [2], [16]–[18], for this reason their results *cannot* be presented in dependence on such a ‘
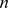
’. Lastly, we present the network properties of CS graphs as a function of 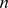 to provide a constant reminder to the reader about the origin of these networks, which is notably distinct.

Over the last decades, there have been many suggestions to define the complexity of one dimensional symbol sequences [44], [48], [60]–[67]. However, none of these methods can be considered as gold standard for all types of applications. For this reason, it does not surprise that the quantification of the complexity of networks, which apparently form more intricate objects than one dimensional symbol sequences, is even less well developed [40]. For this reason, we did not attempt to compare different network complexity measures with each other in order to identify the ‘best’ one, but selected pragmatically the *edge reduction* [47] as a feasible measure to study structural characteristics of networks quantitatively. However, it would be interesting for a future study to investigate the transition of complexity from the sequence level, on which our construction procedure of CS graphs is based, to the network level. Due to the established complexity on the sequence (*step sequence*) and the network (CS graph) level, our construction procedure seems to *conserve* complexity. However, a quantification of this observation could be instructive.

Another interesting aspect of the present paper that deserves more attention in future studies is the general building principle of our growing network model. To our knowledge, this procedure has not been systematically studied yet, despite the fact that it forms such a distinct mechanism compared to many other growing network models, e.g., random or scale-free networks [2], [16]–[18]. This would also be interesting from a biological point of view because our growing network model has been inspired by the biological processes of gene regulation leading to the formation of different types of gene networks [29], [30], [68]. Hence, such a growing network model appears from a biological perspective very natural.

## References

[1] Barabási AL, Oltvai ZN (2004) Network biology: Understanding the cell's functional organization. Nature Reviews 5: 101–113. 10.1038/nrg1272 14735121

[2] Erdös P, Rényi A (1959) On random graphs. I. Publicationes Mathematicae 6: 290–297.

[3] Kolaczyk E (2009) Statistical Analysis of Network Data: Methods and Models. New York: Springer.

[4] Newman M (2010) Networks: An Introduction. Oxford: Oxford University Press.

[5] Watts D, Strogatz S (1998) Collective dynamics of ‘small-world’ networks. Nature 393: 440–442. 9623998 10.1038/30918

[6] Wasserman S, Faust K (1994) Social Network Analysis. Cambridge; New York: Cambridge University Press.

[7] Solomonoff R, Rapoport A (1951) Connectivity of random nets. Bulletin of Mathematical Biophysics 13: 107–117.

[8] Albert R, Barabasi A (2002) Statistical mechanics of complex networks. Rev of Modern Physics 74: 47.

[9] Newman MEJ (2003) The structure and function of complex networks. SIAM Review 45: 167–256.

[10] Bornholdt S, Schuster H, editors (2003) Handbook of Graphs and Networks: From the Genome to the Internet. Wiley-VCH.

[11] Albert R (2005) Scale-free networks in cell biology. Journal of Cell Science 118: 4947–4957. 16254242 10.1242/jcs.02714

[12] Emmert-Streib F, Dehmer M (2011) Networks for Systems Biology: Conceptual Connection of Data and Function. IET Systems Biology 5: 185. 21639592 10.1049/iet-syb.2010.0025

[13] Iguchi K, Kinoshita S, Yamada H (2007) Boolean dynamics of kauffman models with a scale-free network. J Theor Biol 247: 138–51. 17408697 10.1016/j.jtbi.2007.02.010

[14] Crandall R (1978) On the ‘3x+1’ problem. Math Comput 32: 1281–1292.

[15] Lagarias J (1985) The 3n+1 problem and its generalizations. The American Mathematical Monthly 92: 3–23.

[16] Barabási AL, Albert R (1999) Emergence of scaling in random networks. Science 206: 509–512. 10.1126/science.286.5439.509 10521342

[17] Dorogovtesev S, Mendes J (2003) Evolution of Networks: From Biological Nets to the Internet and WWW.Oxford University Press.

[18] Durrett R (2006) Random Graph Dynamics. Cambridge; New York: Cambridge University Press.

[19] Donner RV, Zou Y, Donges JF, Marwan N, Kurths J (2010) Recurrence networksa novel paradigm for nonlinear time series analysis. New Journal of Physics 12: 033025.

[20] Emmert-Streib F, Dehmer M (2010) Influence of the Time Scale on the Construction of Financial Networks. PLoS ONE 5: e12884. 20949124 10.1371/journal.pone.0012884 PMC2948017

[21] Emmert-Streib F (2011) Parametric construction of episode networks from pseudoperiodic time series based on mutual information. PLoS ONE 6: e27733. 22216086 10.1371/journal.pone.0027733 PMC3245224

[22] Lacasa L, Luque B, Ballesteros F, Luque J, Nuno JC (2008) From time series to complex networks: The visibility graph. Proceedings of the National Academy of Sciences 105: 4972–4975. 10.1073/pnas.0709247105 PMC2278201 18362361

[23] Luque B, Lacasa L, Ballesteros F, Luque J (2009) Horizontal visibility graphs: Exact results for random time series. Phys Rev E 80: 046103. 10.1103/PhysRevE.80.046103 19905386

[24] Zhang J, Small M (2006) Complex Network from Pseudoperiodic Time Series: Topology versus Dynamics. Phys Rev Lett 96: 238701. 16803415 10.1103/PhysRevLett.96.238701

[25] Chandra AK, Dasgupta S (2005) A small world network of prime numbers. Physica A: Statistical Mechanics and its Applications 357: 436–446.

[26] Corso G (2004) Families and clustering in a natural numbers network. Phys Rev E 69: 036106. 10.1103/PhysRevE.69.036106 15089360

[27] Oliveira e Silva T (2010) Empirical verification of the $3x\plus 1$ and related conjectures. In: Lagarias JC, editor, The Ultimate Challenge: The $3x\plus 1$ Problem, Providence, Rhosland, USA: American Mathematical Society. 189–207.

[28] Emmert-Streib F, Glazko G (2011) Network Biology: A direct approach to study biological function. Wiley Interdiscip Rev Syst Biol Med 3: 379–391. 21197659 10.1002/wsbm.134

[29] Palsson B (2006) Systems Biology. Cambridge; New York: Cambridge University Press.

[30] Zhang A (2009) Protein Interaction Networks: Computational Analysis. Cambridge; New York: Cambridge University Press.

[31] Ebeling W, Feistel R, Herzel H (1987) Dynamics and complexity of biomolecules. Physica Scripta 35: 761.

[32] Li W (1997) The complexity of dna. Complexity 3: 33–38.

[33] Lagarias J (2003) The 3x+1 problem: An annotated bibliography (1963–1999). ArXiv:math/0309224v13.

[34] Lagarias J (2006) The 3x+1 problem: An annotated bibliography, ii (2000–2009). ArXiv:math/0608208v5.

[35] Ebeling W, Neiman A (1995) Long-range correlations between letters and sentences in texts. Physica A: Statistical and Theoretical Physics 215: 233–241.

[36] Stanley H, Buldyrev S, Goldberger A, Goldberger Z, Havlin S, et al (1994) Statistical mechanics in biology: how ubiquitous are long-range correlations? Physica A: Statistical Mechanics and its Applications 205: 214–253. 11541307 10.1016/0378-4371(94)90502-9

[37] Claussen JC (2007) Offdiagonal complexity: A computationally quick complexity measure for graphs and networks. Physica A: Statistical Mechanics and its Applications 375: 365–373.

[38] Dehmer M, Mowshowitz A (2011) A history of graph entropy measures. Information Sciences 1: 57–78.

[39] Dehmer M, Barbarini N, Varmuza K, Graber A (2009) A large scale analysis of information-theoretic network complexity measures using chemical structures. PLoS ONE 4: e8057. 20016828 10.1371/journal.pone.0008057 PMC2790089

[40] Emmert-Streib F, Dehmer M (2012) Exploring statistical and population aspects of network complexity. PLoS ONE 7: e34523. 22590495 10.1371/journal.pone.0034523 PMC3348134

[41] Kim J, Wilhelm T (2008) What is a complex graph? Physica A: Statistical Mechanics and its Applications 387: 2637–2652.

[42] Mowshowitz A (1968) Entropy and the complexity of graphs: I. an index of the relative complexity of a graph. Bulletin of Mathematical Biophysics 30: 175–204. 5666816 10.1007/BF02476948

[43] Trucco E (1956) A note on the information content of graphs. Bulletin of Mathematical Biophysics 18: 129–135.

[44] Kolmogorov AN (1965) Three approaches to the quantitative definition of ‘information’. Problems of Information Transmission 1: 1–7.

[45] Li M, Vitányi P (1997) An Introduction to Kolmogorov Complexity and Its Applications. Springer.

[46] Solomonoff R (1960) A preliminary report on a general theory of inductive inference. Technical Report V-131, Zator Co., Cambridge, Ma.

[47] Royer L, Reimann M, Andreopoulos B, Schroeder M (2008) Unraveling protein networks with power graph analysis. PLoS Comput Biol 4: e1000108. 18617988 10.1371/journal.pcbi.1000108 PMC2424176

[48] Badii R, Politi A (1997) Complexity: Hierarchical Structures and Scaling in Physics. Cambridge University Press, Cambridge.

[49] Mandelbrot BB (1982) The Fractal Geometry of Nature. San Francisco: WH Freeman.

[50] Harrell FE (2001) Regression Modeling Strategies. New York, NY USA: Springer.

[51] Pearl R, Reed LJ (1920) On the rate of growth of the population of the united states since 1790 and its mathematical representation. Proceedings of the National Academy of Sciences 6: 275–288. 10.1073/pnas.6.6.275 PMC1084522 16576496

[52] Grassberger P (1989) Problems in quantifying self-generated complexity. Helvetica Physica Acta 62: 489–511.

[53] Watts D (1999) Small Worlds: The Dynamics of Networks between Order and Randomness. Princeton University Press.

[54] Jeong H, Tombor B, Albert R, Olivai Z, Barabasi A (2000) The large-scale organization of metabolic networks. Nature 407: 651–654. 11034217 10.1038/35036627

[55] Humphries MD, Gurney K (2008) Network ‘Small-World-Ness’: A Quantitative Method for Determining Canonical Network Equivalence. PLoS ONE 3: e0002051. 18446219 10.1371/journal.pone.0002051 PMC2323569

[56] Kaiser M, Hilgetag CC (2006) Nonoptimal component placement, but short processing paths, due to long-distance projections in neural systems. PLoS Comput Biol 2: e95. 16848638 10.1371/journal.pcbi.0020095 PMC1513269

[57] Crutchfield JP (2012) Between order and chaos. Nat Phys 8: 17–24.

[58] Langton C (1990) Computation at the edge of choas: phase transitions and emergent computation. Physica D 42: 12–37.

[59] Ribeiro AS, Kauffman SA, Lloyd-Price J, Samuelsson B, Socolar JES (2008) Mutual information in random boolean models of regulatory networks. Phys Rev E 77: 011901. 10.1103/PhysRevE.77.011901 18351870

[60] Bialek W, Nemenman I, Tishby N (2001) Predictability, complexity, and learning. Neural Computation 13: 2409–2463. 11674845 10.1162/089976601753195969

[61] Bennett C (1988) Logical depth and physical complexity. In: Herken R, editor, The Universal Turing Machine– a Half-Century Survey, Oxford University Press. 227–257.

[62] Crutchfield JP, Young K (1989) Inferring statistical complexity. Phys Rev Lett 63: 105–108. 10040781 10.1103/PhysRevLett.63.105

[63] Emmert-Streib F (2010) Statistic Complexity: Combining Kolmogorov Complexity with an Ensemble Approach. PLoS ONE 5: e12256. 20865047 10.1371/journal.pone.0012256 PMC2928735

[64] Gell-Mann M, Lloyd S (1998) Information measures, effective complexity, and total information. Complexity 2: 44–52.

[65] Grassberger P (1986) Toward a quantitative theory of self-generated complexity. Int J Theor Phys 25: 907–938.

[66] Lloyd S, Pagels H (1988) Complexity as thermodynamic depth. Annals of Physics 188: 186–213.

[67] Zurek W, editor (1990) Complexity, Entropy and the Physics of Information. Addison-Wesley, Redwood City.

[68] Dehmer M, Emmert-Streib F, Graber A, Salvador A, editors (2011) Applied Statistics for Network Biology: Methods for Systems Biology. Weinheim: Wiley-Blackwell.

